# Case Study of Somaclonal Variation in Resistance Genes* Mlo* and* Pme3* in Flaxseed (*Linum usitatissimum* L.) Induced by Nanoparticles

**DOI:** 10.1155/2017/1676874

**Published:** 2017-02-23

**Authors:** Inese Kokina, Ilona Mickeviča, Marija Jermaļonoka, Linda Bankovska, Vjačeslavs Gerbreders, Andrejs Ogurcovs, Inese Jahundoviča

**Affiliations:** ^1^Department of Biotechnology, Daugavpils University, Institute of Life Sciences and Technology, Laboratory of Genomics and Biotechnology, Parades Street 1A, Daugavpils LV-5401, Latvia; ^2^Department of Technology, Daugavpils University, Institute of Life Sciences and Technology, G. Liberts' Center of Innovative Microscopy, Parades street 1A, Daugavpils LV-5401, Latvia

## Abstract

Nanoparticles influence on genome is investigated worldwide. The appearance of somaclonal variation is a cause of great concern for any micropropagation system. Somaclonal variation describes the tissue-culture-induced phenotypic and genotypic variations. This paper shows the results of somaclonal variation in two resistance genes pectin methylesterase and Mlo-like protein in all tissue culture development stages, as donor plant, calluses, and regenerants of* Linum usitatissimum* induced by gold and silver nanoparticles. In this paper, it was essential to obtain DNA material from all tissue culture development stages from one donor plant to record changes in each nucleotide sequence. Gene region specific primers were developed for resistance genes such as* Mlo *and* Pme3* to define the genetic variability in tissue culture of* L. usitatissimum*. In recent years, utilization of gold and silver nanoparticles in tissue culture is increased and the mechanisms of changes in genome induced by nanoparticles still remain unclear. Obtained data show the somaclonal variation increase in calluses obtained from one donor plant and grown on medium supplemented by gold nanoparticles (*Mlo *14.68 ± 0.98;* Pme3 *2.07 ± 0.87) or silver nanoparticles (*Mlo *12.01 ± 0.43;* Pme3 *10.04 ± 0.46) and decrease in regenerants. Morphological parameters of calluses showed a number of differences between each investigated culture group.

## 1. Introduction

The term somaclonal variation (SV) has been proposed to describe the tissue-culture-induced phenotypic and genotypic variations [1]. The SV is defined as genetic and epigenetic changes between clonal regenerants and the corresponding donor plant [2, 3]. Tissue culture is a reliable tool for plant propagation and evaluation of somaclonal variation what is required to modification of the progeny of clonal propagation. This leads to evolutionary potential of taxon or population to adapt to varying environmental conditions [4, 5]. The regenerated plants may differ genetically or phenotypically from the maternal plant [6].

In this study,* Linum usitatissimum* L. was selected as model plant since it is widely used in fundamental in vitro biotechnological studies due to its unique properties: small genome size, comparatively short life-cycle, and high regeneration ability [7]. Flaxseed is a commercial oilseed that produces triglyceride oil that is a rich source of linolenic acid. Additionally, flax is a source of industrial fibres due to high cellulose content [8, 9]. The SV at the nucleotide sequence of* Linum usitatissimum* L. tissue culture was evaluated in two regions of resistance genes pectin methylesterase* (Pme3)* and powdery mildew resistance gene o* (Mlo)*. MLO proteins were first found in barley, and now they are recognized as a family of plant integral membrane proteins. The plant mutants which do not have wild-type MLO protein show broad spectrum resistance to the powdery mildew; however, they suffer from spontaneous death of cells in response to abiotic stimuli or disorder in development. Thus, it is assumed that these proteins are inhibitors of cell death. The lack of them enhances the probability of starting a cascade of reactions which result in the cell death in plants [10–13]. Mlo genes were investigated in barley, wheat,* Arabidopsis thaliana*, rice, soybean, and rose [14]. However, there are no known investigations concerning Mlo gene in flax cultures. Pectin methylesterase is enzyme affecting pectin which is the main component of plant cell walls in dicotyledonous species. The basal component of pectin is homogalacturonan (HGA) which is a highly methyl-esterified by pectin methyltransferases in regular plant cells, whereas in contrast pectin methylesterases catalyse the specific demethylesterification of HGA within plant cell walls, what can either lead to changes in wall's pH level and targeting of pectin-degrading enzymes, affecting the texture and rigidity alteration of the cell wall. It reveals the crucial role of pectin esterification in plant defence; moreover the pectin structure may determine the result of the interaction between a host and a pathogen [15, 16]. Furthermore,* Pme* genes can play an important role in increasing linseed stem fiber accumulation, which can lead to beneficial properties of linseed [17].

According to the literature, the use of nanotechnologies in in vitro plant systems is rapidly increasing during the last years. Furthermore, the penetrance of nanoparticles (NPs) into cells is described in several studies [18–20]. Gold NPs are widely used in bionanotechnologies because of their unique properties and the possibility to modify their surface with different functional groups. Silver NPs are among the well-studied nanomaterials and represent a special interest as carrier of drug molecules and other bioactive substances [21].

This study characterizes the genetic variability in resistance genes of* L. usitatissimum *donor plant, calluses, and regenerants and morphological changes in calluses of control and experimental groups were documented to evaluate the somaclonal variation.

## 2. Materials and Methods

### 2.1. Synthesis of Nanoparticles

Gold and silver NPs were synthesized in G. Libert's Center of Innovative Microscopy of Daugavpils University, using the methods described by Leung et al. (2004) [22]. The control over the film thickness was done by the method, described in Mecea (2005) [23]. The standard concentration of 500 ppm was used.

### 2.2. Plant Material and Treatment

During this investigation one donor plant of Latvian origin* L. usitatissimum* “Lirina” was used for calluses formation and in vitro cultures were established according to Kokina et al. (2012) [24]. Obtained calluses (*n* = 105) were divided into control group (medium without NPs) and two experimental groups; there media were supplemented by Au or Ag NPs. For each group calluses were induced from 3 explants (leaves) of one donor plant. The number of calluses were equal (*n* = 35) in each experimental and control group. Morphological parameters of calluses were detected under the microscope Eclipse 90i (Nikon, Japan) and stereo microscope AZ 100 (Nikon, Japan).

### 2.3. Sequencing and Specific Primer Design

Genomic DNA was extracted from up to 100 mg of fresh tissues of donor plant leaves, calluses, and regenerants' leaves. Extraction was done with slight modifications for each development stage using the protocol: purification of total DNA from plant tissue (Mini Protocol, DNeasy Plant Mini Kit, Qiagen GmbH, Hilden, Germany). The final elution volume of DNA was 50 *μ*L. The genomic DNA was quantified using a spectrophotometer (NanoDrop 1000, Thermo Scientific, Waltham, USA) to measure absorbance at 260 nm and the purity of DNA was checked. Stock DNA was diluted to make a working solution of 20 ng/*μ*L for further PCR analysis.

PCR and sequencing primers were designed for amplification of the gene regions of* Pme3* and* Mlo* (Table 1). Primers for conventional PCR and sequencing were developed utilizing PyroMark Assay Design software (Qiagen, Germany).

Amplification was carried out in a thermocycler (Veriti 96-Well Thermal Cycler, Applied Biosystems, Foster City, USA) using a Taq PCR Core Kit (Qiagen, Germany). Qiagen Taq DNA polymerase was used in 25 *μ*L reactions with magnesium concentration adjusted to 2.0 mmol/L. The thermocycler was programmed for an initial denaturation of 5 min 94°C, followed by 35 cycles of denaturation for 1 min at 94°C, 1 min of annealing at 51°C, and 30 s of extension at 72°C. The final extension was carried out at 72°C for 10 min with a hold temperature of 4°C at the end. Negative control was included with each round of reactions. PCR products were cleaned-up by ExoSAP-IT (Affymetrix, USA) according to the manufacturer's protocol. Amplified PCR products were sequenced using an ABI 3130xl (Applied Biosystems, USA) capillary sequencer using BigDye Terminator v3.1 chemistry and for purification sequencing products BigDye XTerminator chemistry (Applied Biosystems, USA). Sequencing reactions used the primers designed for sequencing. All sequences were aligned using SeqScape version 2.5 (Applied Biosystems, USA) with default settings. The SV was expressed as the percentage of the differences in the nucleotide sequence in comparison with reference sequence of donor plant.

## 3. Results and Discussion

**Figure 1:**
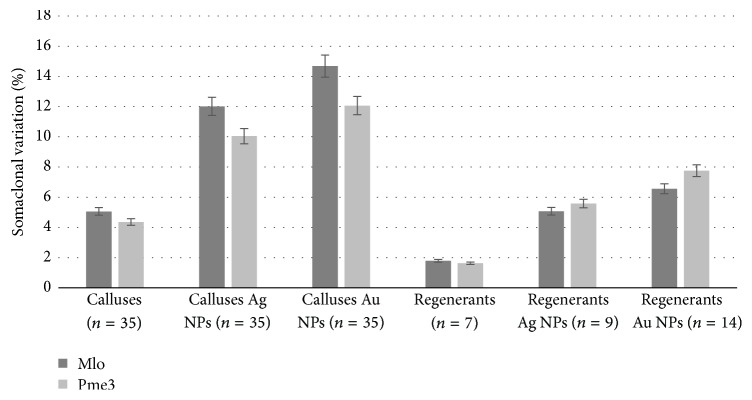
Somaclonal variation in two resistance genes in all development stages of* L. usitatissimum. *Means ± SD and significant difference *P* < 0.05 were indicated, respectively, from the control. The SV was expressed as the percentage of the differences in the nucleotide sequence in comparison with reference sequence of donor plant.

To detect SV at the nucleotide sequence of* Mlo* and* Pme3* resistance genes in* L. usitatissimum* specific primers were developed. PCR reactions were sufficient in all cases and obtained results demonstrate the genetic variability in examined samples (Figure 1). The length of sequence for* Mlo* and* Pme3* was 220 bp and 560 bp, respectively. According to this analysis the genetic variability in investigated gene regions was variable for control and two experimental groups. The highest mean SV (%) was detected in calluses (*n* = 35) grown on medium supplemented by Au NPs (*Mlo* SV = 14.68 ± 0.98;* Pme3* SV = 12.07 ± 0.87), while in calluses (*n* = 35) grown on medium supplemented by Ag NPs (*Mlo* SV = 12.01 ± 0.43;* Pme3* SV = 10.04 ± 0.46) it was lower. The mean SV (%) detected in calluses (*n* = 35) from control group (*Mlo* SV = 5.06 ± 0.09;* Pme3* SV = 4.36 ± 0.13) was the lowest in comparison with experimental groups. The lowest mean SV (%) was observed in regenerants (*n* = 7) in control group (*Mlo* SV = 1.79 ± 0.05;* Pme3* SV = 1.63 ± 0.07). In addition, the mean SV (%) in all analysed regenerants were lower than in calluses from the same group. In regenerants grown on medium supplemented by Au NPs mean SV (%) were (*Mlo* SV = 6.56 ± 0.21;* Pme3* SV = 7.76 ± 0.24) higher than in regenerants supplemented by Ag NPs (*Mlo* SV = 5.07 ± 0.37;* Pme3* SV = 5.58 ± 0.34). It is because embryogenesis is based on obtaining a genetically heterogeneous plant regenerants from callus. Although callus culture consists of undifferentiated fast dividing cells which results in high somaclonal variation level, regenerants from callus culture inherits only part of callus cell's genetic material. This is through the fact that embryo is formed only from one undifferentiated cell from callus culture [25]. No doubt, the number of analysed regenerants in each group was less than the number of analysed calluses, but the major differences in genetic variability were observed in each sequence. In this paper, it was essential to obtain DNA material from all tissue culture development stages from one donor plant leave to record changes in each nucleotide sequence. In this case the equal number of regenerants in each group was impossible to obtain, due to specificity of in vitro culture.

The number of investigations, using molecular biology techniques to detect the somaclonal variation, is based on use of amplified fragment length polymorphisms or intersimple sequence repeats. Such techniques detect unidentified or unspecified mutations and determine either mutation occurred or not [26]. Obtained data confirmed various studies that environment changes induce SV and influence further plant development [3]. The changed environment can be associated with fitness loss [27, 28].

In addition, in tissue culture SV is characterised also by morphological differences [29, 30]. Morphological parameters of calluses showed a number of differences between each investigated culture group (Table 2). The main changes were detected in calluses grown on medium with Au or Ag NPs. They tend to grow in both length and width, forming a spherical shape. The mean number of regeneration zones was variable in all groups. Previous study showed that carbon nanoparticles can increase mean of regeneration zones in flax callus [24]. In present study, the mean number of regeneration zones in control group was the lowest (*n* = 1.24 ± 0.48), although in experimental groups in calluses grown in medium supplemented by Au (*n* = 3.29 ± 1.07) or Ag (*n* = 2.01 ± 0.98) it increased. The calluses of control group showed embryogenesis appearance in 20%, but calluses grown in medium supplemented by Au NPs had 49% of these cells and calluses of medium with Ag NPs had 34%.

## 4. Conclusions

Gained results indicate induction of random changes in the nucleotide sequences of the two resistance gene regions during differentiation of* L. usitatissimum* from callus culture under given in vitro conditions (medium supplemented by Ag NPs or Au NPs). Analyses showed that in vitro culture is instable micropropagation system and in combination with nanotechnologies it could be utilized to produce a better plant yield in different environmental conditions.

## Figures and Tables

**Table 1:** Sequence characteristics of primers developed in this study. PCR and sequencing primers were designed for amplification the gene regions of *Pme3* and *Mlo*.

Primer ID	Primer sequence (5′-3′)	Primer melting temperature	Product length	Accession number
LuMlo-F	CCGATTCTATGGTTTTTTGCAGTC	54°C	226 bp	AJ005341.1
LuMlo-R	GGCAGAAATGGATGAGGAAGAGA	56°C		
LuMlo-S	AAGCTACAAGTGATCATAAC	47°C	220 bp	

LuPme3-F	CCGCTTGAACATAGAAAAACAGCT	56°C	573 bp	AF308812.1
LuPme3-R	TCTTTTTTTTCACGTGGCGACT	55°C		
LuPme3-S	TTTAGCCCAAAATTGAG	44°C	560 bp	

**Table 2:** Results of morphological parameters of calluses of control and experimental groups (*n* = 35). Means ± SD and significant difference at *P* < 0.05 were indicated, respectively, from the control.

Group	Mean calluses width, mm ± SD	Mean calluses length, mm ± SD	Regeneration zones ± SD	Embryogenesis appearance, %
Control	4.18 ± 1.36	6.22 ± 0.67	1.24 ± 0.48	20
AgNPs	5.37 ± 3.47	7.00 ± 2.54	2.01 ± 0.98	34
AuNPs	6.39 ± 2.82	6.55 ± 2.36	3.29 ± 1.07	49
